# Recommendations for the Design and Implementation of Virtual Reality for Acquired Brain Injury Rehabilitation: Systematic Review

**DOI:** 10.2196/26344

**Published:** 2021-07-30

**Authors:** Sophie Brassel, Emma Power, Andrew Campbell, Melissa Brunner, Leanne Togher

**Affiliations:** 1 Discipline of Speech Pathology, Sydney School of Health Sciences Faculty of Medicine and Health The University of Sydney Sydney Australia; 2 Speech Pathology Graduate School of Health University of Technology Sydney Sydney Australia; 3 Cyberpsychology Research Group Faculty of Medicine and Health The University of Sydney Sydney Australia

**Keywords:** virtual reality, acquired brain injury, traumatic brain injury, rehabilitation, systematic review, recommendations, cognitive communication, mobile phone

## Abstract

**Background:**

Virtual reality (VR) is increasingly being used for the assessment and treatment of impairments arising from acquired brain injuries (ABIs) due to perceived benefits over traditional methods. However, no tailored options exist for the design and implementation of VR for ABI rehabilitation and, more specifically, traumatic brain injury (TBI) rehabilitation. In addition, the evidence base lacks systematic reviews of immersive VR use for TBI rehabilitation. Recommendations for this population are important because of the many complex and diverse impairments that individuals can experience.

**Objective:**

This study aims to conduct a two-part systematic review to identify and synthesize existing recommendations for designing and implementing therapeutic VR for ABI rehabilitation, including TBI, and to identify current evidence for using immersive VR for TBI assessment and treatment and to map the degree to which this literature includes recommendations for VR design and implementation.

**Methods:**

This review was guided by PRISMA (Preferred Reporting Items for Systematic Reviews and Meta-Analyses). A comprehensive search of 11 databases and gray literature was conducted in August 2019 and repeated in June 2020. Studies were included if they met relevant search terms, were peer-reviewed, were written in English, and were published between 2009 and 2020. Studies were reviewed to determine the level of evidence and methodological quality. For the first part, qualitative data were synthesized and categorized via meta-synthesis. For the second part, findings were analyzed and synthesized descriptively owing to the heterogeneity of data extracted from the included studies.

**Results:**

In the first part, a total of 14 papers met the inclusion criteria. Recommendations for VR design and implementation were not specific to TBI but rather to stroke or ABI rehabilitation more broadly. The synthesis and analysis of data resulted in three key phases and nine categories of recommendations for designing and implementing VR for ABI rehabilitation. In the second part, 5 studies met the inclusion criteria. A total of 2 studies reported on VR for assessment and three for treatment. Studies were varied in terms of therapeutic targets, VR tasks, and outcome measures. VR was used to assess or treat impairments in cognition, balance, and anxiety, with positive outcomes. However, the levels of evidence, methodological quality, and inclusion of recommendations for VR design and implementation were poor.

**Conclusions:**

There is limited research on the use of immersive VR for TBI rehabilitation. Few studies have been conducted, and there is limited inclusion of recommendations for therapeutic VR design and implementation. Future research in ABI rehabilitation should consider a stepwise approach to VR development, from early co-design studies with end users to larger controlled trials. A list of recommendations is offered to provide guidance and a more consistent model to advance clinical research in this area.

## Introduction

### Background

The use of virtual reality (VR) in health care has expanded in recent years and continues to be investigated due to the increasing availability and advancement of technology [1,2]. VR is being used in clinical research for the assessment and therapeutic intervention of impairments associated with acquired brain injuries (ABIs), which is an umbrella term for brain injuries that are sustained after birth [3,4]. A considerable evidence base exists for using VR for ABI rehabilitation, with a particular focus on stroke [5-14]. Traumatic brain injury (TBI) is another ABI that is increasingly being investigated with VR technologies; however, the evidence base is smaller and not as rigorous as that for other ABIs. TBI leads to alterations in brain function and pathology caused by a blow or other external force on the head [15]. As a major cause of disability and mortality, ABIs are increasingly being considered as a public health burden and place significant economic strain on society [16]. People who sustain an ABI can experience physical, cognitive, and communication impairments that are often long lasting and significantly impact their everyday functioning [3,17-20].

VR refers to “a computer-generated digital environment that can be experienced and interacted with as if that environment were real” [21]. VR systems are typically classified as immersive, semi-immersive, or nonimmersive [22,23], with immersion referring to the level of user perception with regard to being in a virtual environment (VE) rather than the real world [24]. Immersive VR systems supply VEs with a changing field of view via head-mounted displays (HMDs) [22,23,25]. Movement within immersive VEs is achieved via hardware such as head trackers, hand controllers, and body motion sensors [22,23]. Semi-immersive VR refers to systems that use projection-based systems (eg, driving simulators and use of shutter glasses), whereas nonimmersive VR systems include basic desktop displays and videogames [22,23,25]. To improve the delivery of assessment, treatment, and clinical outcomes for people with ABI, the use of VR should be further explored because of its potential to address limitations and produce benefits over conventional assessment and treatment methods [26-29].

### Recommendations for VR Design and Implementation in ABI Rehabilitation

The existing literature provides guidance for safety and ethical considerations in clinical VR research [30-33], although there is a general lack of focus on design and development processes for using VR in ABI rehabilitation [34,35]. Some prior studies have proposed useful recommendations for implementing VR in health care and rehabilitation research; however, they are often specific to a particular VR system or do not focus on ABI [36-38]. Given the potentially limited applicability of these recommendations for designing and implementing VR in ABI rehabilitation, developing a set of recommendations would be beneficial for guiding research in this field.

Part 1 of this review had originally planned to include recommendations for using VR in TBI rehabilitation exclusively; however, no studies were identified. Examining the use of VR with other ABIs may provide guidance for this population. Recommendations specific to ABI are necessary, as individuals may experience motor, visual, or vestibular impairments that could impact their ability to use VR [39,40]. There are studies that propose recommendations for using VR in ABI rehabilitation [35,41]; however, there are no known systematic reviews on this topic. Developing recommendations based on existing studies and frameworks would be valuable for determining critical technological factors to guide the design and implementation of immersive VR in ABI rehabilitation.

### VR for ABI Rehabilitation

ABI rehabilitation aims to improve function or provide compensatory strategies to reduce impairments and increase participation in activities and quality of life [42-45]. Goals and opportunities to practice real-life, meaningful tasks should be provided to maximize function and enable participation outside of clinical settings [43,46]. Examples include practicing cognitive, physical, or communication therapy goals in everyday activities [47,48] or relevant community settings (eg, home, cafes, and work) [49,50]. Early and intensive therapy is also recommended [51-53]. This focus on generalization and intensity can support neuroplastic changes, which in turn can assist with functional recovery following ABIs [51,54]. Furthermore, treatment programs should be goal oriented [53] and tailored to individual needs [55]; however, this can be challenging due to the complexity and diversity of ABIs [19,44]. Other limitations of the current assessment and treatment approaches in ABI rehabilitation include difficulties providing sufficient intensive therapy to allow for neuroplastic change or provision of services to patients residing in rural areas [44,56]. Advances in technology have provided opportunities for researchers to investigate the use of VR as a novel rehabilitation tool to overcome some of these barriers [29,56].

The benefits of VR for ABI rehabilitation include enhanced ecological validity, the ability to maintain experimental control over assessment and treatment standardization [28,57-59], and the option to cater to individual skills and goals by controlling task complexity [59,60]. VR can also provide relatively naturalistic VEs [28,61] for repeated practice of functional tasks such as activities of daily living [28,58,62], which may assist with generalizing targeted skills [63]. VR can also enhance patient motivation [61,64], which is necessary for neurorehabilitation, as repeated practice is required to achieve adequate treatment outcomes. Furthermore, VR may reduce barriers to accessing rehabilitation services, such as affordability and geographical isolation [56].

The development of VR for ABI rehabilitation should incorporate co-design design principles [65], which is lacking in the current literature, especially for TBI. Co-design engages intended users (eg, patients and therapists) in the design and development of products, including VR [21]. People with ABI can experience complex and debilitating impairments. By determining their specific needs and capabilities, VR systems can be developed to meet patient and therapist needs, improve success in clinical practice, and maximize therapeutic outcomes [21,40,66,67].

With regard to using VR in TBI rehabilitation specifically, there are no known systematic reviews that examine the evidence base for using immersive VR to assess and treat any impairment sustained from TBIs. Experimental and review studies have mainly investigated VR for assessing or treating cognitive or motor impairments [34,56,57,68-72]. This current evidence base provides some support for using VR for TBI rehabilitation; however, the quality of the evidence is relatively low [34], and many studies include nonimmersive and semi-immersive systems [45,73-77] rather than focusing on immersive VR technology with HMDs. Issues identified in experimental studies include heterogeneity (eg, severity of TBI, VR system used, and outcome measures) with small sample sizes and a lack of randomized controlled trials (RCTs), resulting in the inability to perform meta-analysis [68,71]. Existing reviews provide important information; however, many reviews are not systematic in design or lack quality appraisal of included studies [34,45,56,57,70], focus on VR for only cognitive or motor impairments [68-72], do not consider immersive VR only [45,56,57,68-72,78], or do not review both assessment and treatment studies [56,57,68,69,72,78]. As literature to date has not focused on using immersive VR across the clinical spectrum of TBI rehabilitation, this review aims to identify and evaluate the use of immersive VR for the assessment and treatment of any impairment sustained within this group.

### Objectives

This systematic review contains two parts and aims to:

1. Identify and synthesize existing recommendations and frameworks for designing and implementing therapeutic VR for ABI rehabilitation. By doing so, we aim to identify key technological and co-design factors to propose recommendations for the systematic development of VR apps in this field.
2. Determine the current published evidence base for using immersive VR for TBI assessment and treatment. The identified studies will be compared against the synthesized recommendations from part 1 to determine strengths and potential gaps in the literature with reference to recommendations for VR design and implementation to propose ways to improve future research and practice.

## Methods

### Protocol and Registration

This review has been registered with the International Prospective Register of Systematic Reviews (CRD42020152884) and was guided by the PRISMA (Preferred Reporting Items for Systematic Reviews and Meta-Analyses) Statement [79].

### Search Strategy

A systematic search was conducted in August 2019. A total of 11 databases were accessed: CINAHL, Cochrane Central, Embase, Institute of Electrical and Electronics Engineers Xplore, MEDLINE, ProQuest Central, PsycBITE, PsychINFO, Scopus, speechBITE, and Web of Science. Search strategies were adapted for individual database requirements. Gray literature was also searched to ensure that all relevant studies were identified (ie, peer-reviewed conference proceedings and clinical guidelines). Additional studies were sourced by hand searching the reference lists of the included papers and repeating database searches in June 2020.

Two systematic searches were conducted to address the research aims in this review. For part 1, the general search strategy was *virtual reality* AND *assessment* OR *intervention* OR *research* AND *recommendations* AND *neurorehabilitation* OR *brain injury*. For part 2, the general search terms were *virtual reality* AND *traumatic brain injury* AND *assessment* OR *intervention*. The following limits were placed on the searches and studies for inclusion: (1) peer-reviewed, (2) full-text availability, (3) written in English, and (4) published between 2009 and 2020. Literature from the past 10 years was included to reflect current research, as the use of VR in health care and rehabilitation is changing rapidly. A detailed search strategy is provided in Multimedia Appendix 1, and the inclusion and exclusion criteria are provided in Textbox 1.

**Inclusion and exclusion criteria for included studies.**

**Inclusion and Exclusion Criteria**

**Part 1**

- Provided clear guidelines, consensus statements, recommendations, considerations, or pathways for using virtual reality with adults aged ≥18 years with an acquired brain injury, or the study referred to acquired brain injury populations.
- All study designs were considered.
- Included data or review of existing scientific evidence as a basis for recommendations.
- All virtual reality types were considered (recommendations for development and implementation are likely to be applicable for all therapeutic virtual reality designs despite potential variability in virtual reality systems and levels of immersion [66]).
- Papers that provided recommendations for a specific virtual reality system were excluded (ie, recommendations could not be applied to using virtual reality for acquired brain injury rehabilitation more broadly).

**Part 2**

- Included adults aged ≥18 years with a diagnosis of traumatic brain injury (studies were required to have ≥50% participants with a traumatic brain injury).
- Evaluated use of immersive virtual reality for assessing or treating any impairment sustained from a traumatic brain injury (immersive virtual reality was considered due to rapid advancements in technology [80]).
- Intervention studies included pre-post outcomes.
- Original research design (eg, randomized controlled trial, case series, and case study).
- Review papers and studies with semi-immersive or nonimmersive virtual reality systems were excluded.

### Screening Process

The following process was conducted separately for each systematic search. Search results were exported to a reference manager (EndNote X9, Clarivate), where any duplicate references were excluded. Nonduplicate references were exported to a systematic review management program (Covidence) [81] to review titles and abstracts against search terms and eligibility criteria. An independent reviewer completed the reliability screening for 25.04% (426/1701) of randomly selected nonduplicate references from both searches. All disagreements were resolved via discussion. For part 1, the interrater point-by-point agreement was 97.6% (243/249), Cohen κ was 0.733, and 95% CI was 0.598-0.948, indicating substantial agreement [82]. For part 2, the interrater point-by-point agreement was 91.5% (162/177), Cohen κ was 0.605, and 95% CI was 0.426-0.785, indicating substantial agreement [82]. Full texts of eligible papers were retrieved and assessed for inclusion following this process.

### Data Extraction

The following data were extracted and entered into a Microsoft Excel [83] spreadsheet by the first author. Part 1 included bibliographic data, study design and level of evidence, population, focus of the paper, and VR recommendations. Part 2 included bibliographic data, study design and level of evidence, participant characteristics, VR system and equipment, VR task, dosage and time in VR, outcome measures, results, adverse effects, inclusion and exclusion criteria, and evaluation against recommendations synthesized from studies in part 1.

### Data Synthesis and Analysis

For part 1, data were synthesized via a qualitative meta-synthesis: (1) extracting recommendations from the included studies, (2) coding individual recommendations, (3) grouping recommendations based on similarities, and (4) synthesis of grouped recommendations to produce a single comprehensive list [84]*.* For part 2, meta-analysis was not performed due to the heterogeneity of the included studies. Therefore, the results are presented descriptively and in summary tables.

### Quality Assessment

Where possible, studies were classified according to the Oxford Centre of Evidence-Based Medicine Levels of Evidence [85]. This hierarchy classifies evidence according to research methodology from the highest (level 1, systematic reviews) to the lowest (level 5, mechanism-based reasoning) levels of evidence. Methodological quality was assessed using appraisal tools relevant to study design [86]: A Measurement Tool to Assess Systematic Reviews for systematic reviews [87]; Joanna Briggs Institute (JBI) checklists for case series [88], case studies [88], qualitative studies [89], and papers of text and opinion (eg, consensus, expert opinion, and perspectives) [90]; and the Mixed Methods Appraisal Tool for mixed methods studies [91]. The PRISMA extension reporting guideline was used for scoping reviews [92], as no suitable appraisal tools were found. The first author and an independent rater conducted the quality assessment. Any disagreements were resolved through consensus with the fourth author. Studies were not excluded based on appraisal tool scores. The scores were used to guide the interpretation of the results.

## Results

### Part 1

Part 1 of this review aimed to develop recommendations for the design and implementation of therapeutic VR for ABI rehabilitation based on a synthesis of the existing literature.

#### Study Selection

**Figure 1:**
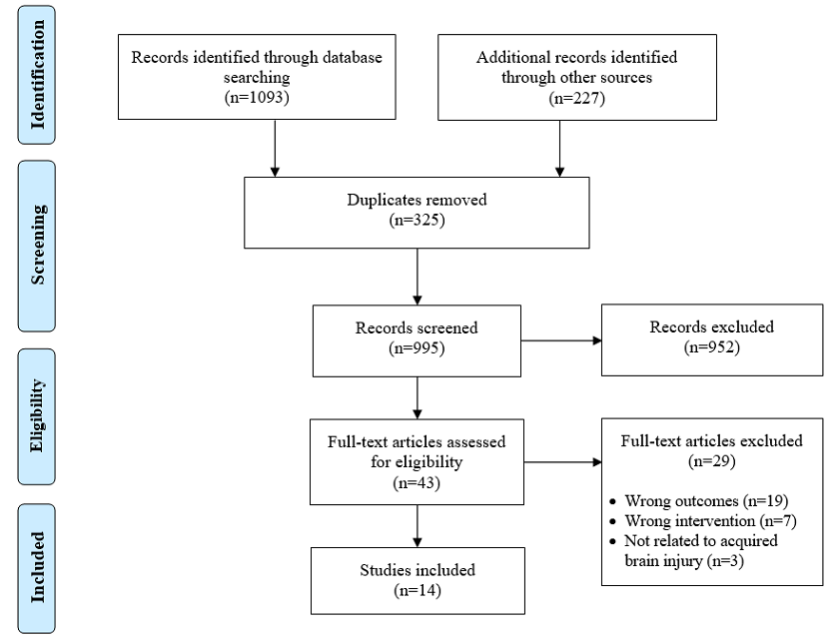
PRISMA flow diagram for studies included in part 1.

Database, gray literature, and hand searches returned 1320 potential studies. Following the removal of duplicates, 995 studies were reviewed for keywords and eligibility criteria. After reading the full texts, 14 studies met the criteria for this review (Figure 1).

#### Study Design and Level of Evidence

A variety of study designs were included: 1 systematic review [10], 4 literature reviews [93-96], 5 text and opinion papers [39-41,66,67], 2 mixed methods studies [97,98], 1 scoping review [35], and 1 qualitative study [99]. A total of 5 of these studies were published as conference proceedings [39,93,96,98,99]. Further information regarding the details of the included studies is presented in Table 1.

**Table 1:** Study characteristics of the papers included in part 1.

Author	Country	Study design	Population or participant details	VR^a^ definition (VR equipment and environment)	Aims of the study
Birckhead et al [66]	United States	Expert opinion consensus	Clinical health care and rehabilitation (makes references to stroke)	Immersive VR, defined VR as using an “HMD^b^ with a close proximity screen”	To develop a methodological, best practice framework to guide development and implementation of high-quality therapeutic VR in health care
Bryant et al [99]	Australia	Qualitative^c^	Rehabilitation, including ABI^d^ (communication disability)	Immersive VR	To explore views of health care and VR professionals on VR-based rehabilitation
Deutsch and Westcott McCoy [93]	United States	Literature review^c^	Neurological rehabilitation	Mentions nonimmersive, semi-immersive, and immersive VR systems	To review literature on VR in neurorehabilitation and offer suggestions for bridging gaps between research and practice when adopting VR
Glegg and Levac [39]	Canada	Perspective or discussion^c^	Rehabilitation (based on neurorehabilitation)	Mentions nonimmersive, semi-immersive, and immersive VR systems	To provide recommendations for the development, research, and clinical implementation of VR based on known barriers and facilitators
Glegg and Levac [35]	Canada	Scoping review	Rehabilitation (including ABI)	Included studies used a range of nonimmersive, semi-immersive, and immersive VR systems	To determine factors that contribute to facilitators and barriers to implementing VR in rehabilitation and to develop recommendations to address barriers
Kellmeyer [40]	Germany	Perspective or discussion	Neurology and psychiatry	Immersive VR	To discuss implications of using highly immersive VR systems within neurology and psychiatry, including ethical issues and adverse effects
Laver et al [10]	Australia	Systematic review (1^e^)	Stroke	Included studies used a range of nonimmersive, semi-immersive, and immersive VR systems	To determine efficacy of VR for stroke rehabilitation
Lee et al [97]	Korea	Mixed methods	Acute stroke; 8 participants (4 male and 4 female; mean age 63 years)	Semi-immersive VR system (Microsoft Kinect; whack-a-mole game for upper limb movement)	To explore patients’ perceived difficulty and enjoyment during VR rehabilitation and the factors affecting experiences and to suggest implementation strategies for VR-based rehabilitation for acute stroke
Levin et al [94]	Canada	Literature review	ABI (upper limb impairments)	Defines VR with examples of nonimmersive, semi-immersive, and immersive systems	To review motor control and learning principles and to discuss how they can be included in the design of VR training environments
Lewis and Rosie [95]	New Zealand	Literature review	Chronic neurological conditions (associated movement disorders)	Included studies used a range of nonimmersive, semi-immersive, and immersive VR systems	To review studies that examine users’ responses to VR interventions and develop suggestions for how future VR systems can address user needs and expectations
Proffitt and Lange [41]	United States	Perspective or discussion	Stroke	VR systems that allow for immersion without assistance (ie, robotic devices)	To outline steps for developing VR interventions for stroke rehabilitation
Proffitt et al [67]	United States	Review of case studies	Rehabilitation (including stroke and TBI^f^)	Included studies used a range of nonimmersive and semi-immersive VR systems	To review examples of end user involvement in VR research to provide recommendations for user-engaged design and implementation for VR in clinical practice
Ramírez-Fernández et al [96]	Mexico	Literature review^c^	Stroke (upper limb impairments)	Not specified; all VR environments	To develop a taxonomy of VR design factors for upper limb rehabilitation of stroke patients
Vaezipour et al [98]	Australia	Mixed methods^c^	Speech pathologists trialed a VR system designed for neurological conditions including ABI (communication impairments)	Immersive VR system (HTC VIVE Pro; simulated kitchen activity)	To explore speech pathologists’ perspectives about immersive VR for rehabilitation of neurogenic communication disorders and to determine advantages and barriers to VR use

^a^VR: virtual reality.

^b^HMD: head-mounted display.

^c^Conference proceeding.

^d^ABI: acquired brain injury.

^e^Oxford levels of evidence (not applied to mixed methods or qualitative papers).

^f^TBI: traumatic brain injury.

#### Populations and Participants

Studies included a range of participants or populations: ABIs (8/14, 57%) [39,40,67,93-95,98,99], stroke only (4/14, 29%) [10,41,96,97], or a variety of medical conditions with reference to ABIs (2/14, 14%) [35,66].

#### VR Details

Various VR systems and levels of immersion were considered or described in the included studies: a combination of nonimmersive, semi-immersive, and immersive systems (7/14, 50%) [10,35,39,67,93-95]; immersive systems (4/14, 29%) [40,66,98,99]; semi-immersive systems (1/14, 7%) [97]; any VR environment (1/14, 7%) [96]; and VR systems that provided immersion without robotic devices (1/14, 7%) [41].

#### Qualitative Data Synthesis and Analysis

##### Overview

Three key phases of therapeutic VR development in health care were recommended according to the methodological framework developed by Birckhead et al [66]: (1) content development via end user involvement and iterative testing processes; (2) testing for feasibility, acceptability, tolerability, and efficacy; and (3) conducting RCTs for VR versus control interventions. Similar processes were described by Proffitt and Lange [41] and Laver et al [10], who recommended that initial VR studies for stroke rehabilitation should determine safety, validity, and usability with intended users before conducting larger trials and comparative studies [10,41].

**Figure 2:**
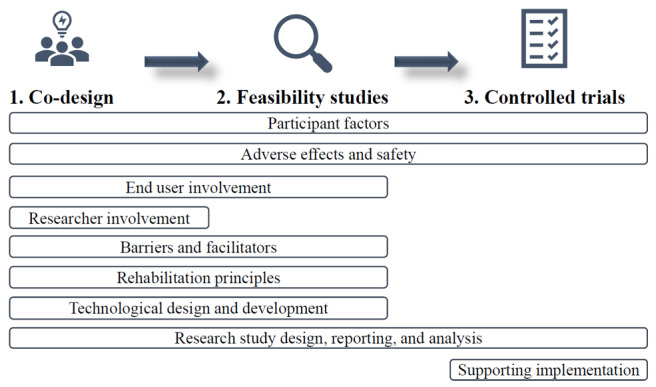
Phases and categories of recommendations for virtual reality design and implementation.

Further synthesis and analysis of recommendations from the 14 included studies identified nine categories of recommendations related to participant, design, and technology factors for VR development and implementation in ABI rehabilitation: (1) end user involvement; (2) participant factors; (3) adverse effects and safety; (4) researcher involvement; (5) barriers and facilitators; (6) rehabilitation principles; (7) technological design and development; (8) supporting implementation; and (9) research study design, reporting, and analysis. Many of these categories are interlinked and can be considered across the suggested phases of design and implementation for therapeutic VR (Figure 2). A summary of the nine categories of recommendations is presented in Textbox 2, and a complete downloadable version is provided in Multimedia Appendix 2 [10,35,39-41,66,67,93-99].

**Summarized recommendations for design and implementation of virtual reality for acquired brain injury rehabilitation.**

**Category and Recommendations**

**End User Involvement**

- Involve end users when designing virtual reality apps [39,40,66,67,95,98].

**Participant Factors**

- Consider participant factors when designing prototypes (eg, age, gender, ethnicity, health conditions, social position, cognition, and physical limitations) [40,66,95-98].
- Determine the impact of virtual reality on motivation and how to sustain engagement [10,66,98].
- Observe users to learn about their behavior [66].

**Adverse Effects and Safety**

- Measure and report physical and emotional adverse effects [40,66].
- Examine safety of virtual reality devices and tasks to determine suitability and contraindications [39,67,98].

**Researcher Involvement**

- Develop ideas and evaluate virtual reality prototypes as a team [66].

**Determining Barriers and Facilitators to Virtual Reality**

- Identify potential barriers and facilitators to designing and implementing virtual reality with key stakeholders [35,39,66,67] and offer solutions or implementation strategies [35].

**Rehabilitation Principles**

- Maintain therapeutic principles in virtual reality tasks (eg, principles of motor learning) [94,95].
- Tasks should be progressively challenging and customizable [94-98].
- When providing feedback, consider real-time knowledge of performance [94-96] and multimodal feedback (eg, visual, auditory, and haptic) [94,96,97].

**Technological Design and Development**

- Use hardware and software that is unrestrictive and allows for movement and possible postural constraints [95].
- Work in collaboration with virtual reality experts, game developers, and engineers [35,39,40].

**Supporting Implementation**

- Support therapists with virtual reality [35,39,67,93,99] and provide continued training [35,39,93].
- Provide information, training, and support for patients using virtual reality [95,96,98,99].

**Research Study Design, Reporting, and Analysis**

- Conduct larger, adequately powered trials [10].
- For randomized controlled trials, use appropriate randomization, conceal allocation, use CONSORT (Consolidated Standards of Reporting Trials) guidelines [66], and justify and describe control conditions [10,66,93].
- When reporting virtual reality research, consider using reporting guidelines (eg, Template for Intervention Description and Replication) [66], describe intervention details [66,67] and efforts to conceal allocation clearly [66], register trials on a publicly accessible registry, and publish all research regardless of outcomes [66].
- When selecting outcome measures, consider clinical relevance and validity [66,67], patient-reported outcomes [66,67], and pre- and postintervention measures [66,94]; measure long-term effects of virtual reality interventions [10,66]; compare against nonrandomized control groups [66]; and evaluate virtual reality in natural environments [93].

##### End User Involvement

Involving end users in co-design for therapeutic VR was recommended in 6 studies [39,40,66,67,95,98]. Suggested end users include therapists and patients who ultimately benefit from the VR systems. Co-design encourages those involved in the design process to gather user feedback to improve the iterations of VR tasks under development. It was recommended that this feedback includes, for example, patient willingness to try VR, what worked or did not work, or which VR systems therapists wish to acquire. Gathering user feedback, both positive and negative, is an important part of co-design because VR prototypes should be iteratively tested by end users and continually refined to better meet patient and therapist needs [66,67].

##### Participant Factors

A range of participant factors should be considered when developing therapeutic VR for ABI rehabilitation [10,40,66,95,96,98]. This is because individuals with neurological impairments may experience physical and cognitive conditions that could impact their understanding and use of VR. In addition, more meaningful and effective VR programs can be developed when specific user needs are considered [40,66,95,97]. Observing patient behavior in a clinical context or conducting surveys and interviews may provide insights into these factors to be considered [66]. Ways to enhance and sustain patient motivation should also be addressed [10,66,98].

##### Adverse Effects and Safety

Participants may experience a range of potential adverse physical and emotional effects when using VR. Some of the potential adverse effects of using VR include headaches, vertigo, nausea, dizziness, fear, and anxiety [66]. A total of 5 of the included studies [39-41,66,98] recommended that these adverse effects should be measured and reported during the development of VR tasks for neurological populations. Measuring adverse effects is necessary to establish a research base for the safety of VR devices and programs and to determine any contraindications for use with people who have an ABI [39-41,98].

##### Determining Barriers and Facilitators

Potential barriers and facilitators of VR use and implementation should be identified via site-specific assessments or interviews during VR development [35,39,66,67]. These barriers and facilitators should be from the perspective of patients and health care providers. Solutions to address the identified barriers should be provided to support successful VR design and implementation [35]. This is particularly important during the design and feasibility testing of therapeutic VR [66].

##### Researcher Involvement in Design

Birckhead et al [66] provided recommendations for researcher involvement and collaboration in the initial design processes. Research teams should develop several ideas for VR tasks and then determine the most feasible and suitable ideas for prototype testing. It is argued that team collaboration is essential for developing therapeutic VR and can often lead to more innovative and improved designs [66].

##### Rehabilitation Principles

A total of 5 studies [94-98] provided recommendations for incorporating rehabilitation principles (eg, principles of motor learning and control [94,100]) when designing VR tasks for ABI rehabilitation. Motor patterns used during VR tasks should provide patients with rehabilitation benefits [95], and tasks should be able to be modified to accommodate impairment severities and stages of recovery [94-98]. Recommendations were also made to provide feedback. Some studies suggested that feedback on performance should be given in real time to engage and motivate patients [94-96]. Levin et al [94] recommended that knowledge of both performance and results should be provided in a way that does not interrupt task progression. Multimodal feedback (eg, visual, auditory, and haptic) should also be considered to potentially improve engagement [94,96]. Additional recommendations included designing tasks that have purposeful goals and providing the opportunity for multiple repetitions of rehabilitation targets [94].

##### Technological Design and Development

A total of 4 of the included studies [35,39,40,95] discussed technological factors to be considered when designing therapeutic VR. Researchers and therapists are recommended to work with game developers and engineers, as they have technological and design expertise to build tasks that meet patients’ and therapists’ needs [35,39,40]. Hardware (eg, HMDs and hand-held devices) and software for VR tasks should also be carefully considered to prevent potential limitations or failures of the VR technology [95]. Examples include designing or selecting systems that allow for adequate movement, providing a sufficient field of view, and reducing the complexity of hardware.

##### Supporting Implementation

Recommendations for supporting the implementation of VR in practice for therapists and patients were provided in 8 of the included studies [35,39,67,93,95,96,98,99]. Recommendations for therapists included providing tailored clinical training packages, providing education about using VR to achieve rehabilitation outcomes, and identifying ways to assist with troubleshooting and implementation [35,39,67,93,99]. Education and training could also be provided to students in relevant professions [39,93]. In terms of supporting patients, therapists should provide adequate information about the purpose of VR and clear instructions for use [98,99] and monitor patient performance regardless of practice settings (eg, rehabilitation units, home-based therapy), as it is necessary for rehabilitation tasks to be performed correctly to achieve sufficient treatment outcomes [95,96].

##### Study Design, Reporting, and Analysis

Among the included studies, 4 [10,41,66,93] proposed recommendations for the design, analysis, and reporting of clinical VR research. Recommendations were related, but not limited, to ensuring rigorous randomization processes in clinical trials, use of reporting guidelines, and detailing specific components of the VR tasks (eg, equipment, therapy dose, and intensity). Recommendations were provided for selecting and using outcome measures to determine the effectiveness of VR interventions [10,41,66,93]. Researchers are encouraged to use outcome measures that are clinically relevant, validated, and standardized, and researchers should also consider using patient-reported measures [66] or target participation outcomes [93]. With regard to the timing of outcome measures, Birckhead et al [66] recommended that measures should be taken at least pre- and postintervention, whereas Laver et al [10] suggested that outcome measures should be taken at least 3 months postintervention to determine long-term effects.

#### Methodological Quality

The methodological quality of the included studies varied (Multimedia Appendix 3 [10,35,39-41,66,67,87-92,97-99, 101-103]). Cross-comparison of study quality could not be made, as quality assessment could not be conducted with a single appraisal tool and no suitable appraisal tools were found for all study types. Studies with relatively high methodological quality included one systematic review [10], papers of text and opinion [39-41,66,67], one qualitative focus group study [99], one scoping review [35], and one mixed methods study [97]. The second mixed methods study [98] met four out of five criteria for quantitative methodology but had limitations in reporting qualitative and mixed methods components. However, this study was presented as a conference proceeding, so all details may not have been included.

#### TBI Guidelines

Published guidelines for the management of TBI were included as gray literature in the search for this review. None of the reviewed guidelines [47,48,50,53,104-110] provided clear recommendations for the development or implementation of VR for TBI rehabilitation. Despite this, three guidelines suggested that VR is a priority area for research for the assessment and management of TBI [53,104,105], highlighting the potential of VR for TBI rehabilitation and the importance of conducting research in this area.

### Part 2

Part 2 of this review aimed to identify current evidence for using immersive VR for assessment and treatment in TBI rehabilitation. These studies were also examined to determine the extent to which they incorporate recommendations for developing and implementing therapeutic VR based on the findings from part 1 of this review.

#### Study Selection

**Figure 3:**
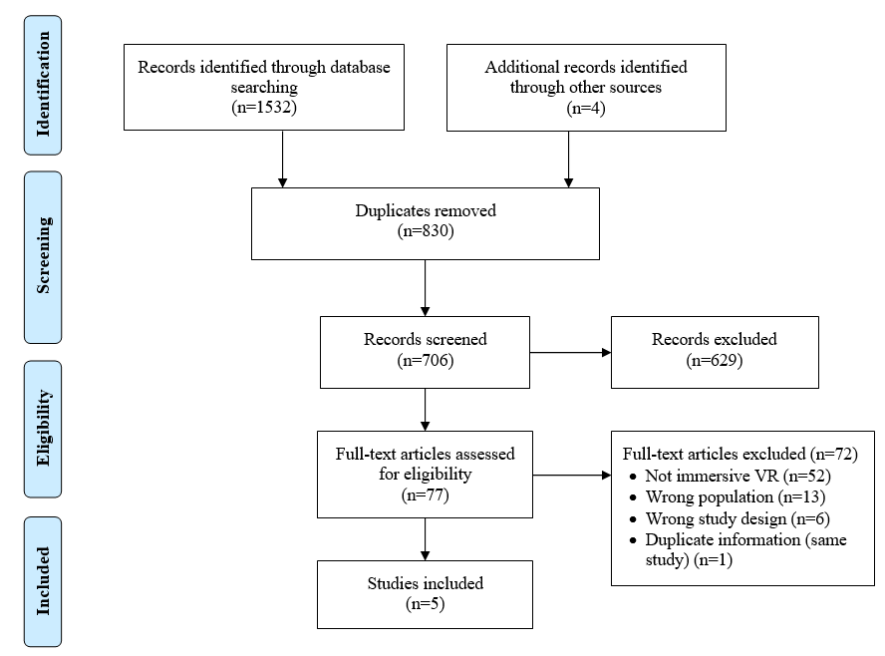
PRISMA flow diagram for studies included in part 2. VR: virtual reality.

Database, gray literature, and hand searches returned 1536 potential studies. A total of 830 duplicate studies were removed. Following the screening of titles and abstracts, 77 nonduplicates were identified for full-text screening. Of these studies, 5 met the inclusion criteria. This process is illustrated in Figure 3.

#### Study Design and Level of Evidence

Included studies investigated the use of VR for assessment (2/5, 40%) [111,112] or treatment (3/5, 60%) [101-103] of impairments following TBI (Table 2). The overall rating of the level of evidence [85] was low. Assessment studies provided level 4 evidence [111,112]. Treatment study designs included 1 case series (level 4) [101] and 2 single case studies [102,103].

**Table 2:** Study characteristics and participant details of the studies included in part 2.

Author	Country	Study design	Participant numbers (TBI^a^ severity)	Age (years)	Gender	Time post TBI	Setting
Banville and Nolin [111]	Canada	Quasi-experimental assessment (4^b^)	TBI=31 (7 moderate and 24 severe) and matched healthy controls=31	TBI: mean 27 (SD 11) and controls: mean 27 (SD 11)	TBI: 23 males and 8 females; controls: 23 males and 8 females	Mean 3.78 (SD 2.5) years	Outpatient
Cikajlo et al [101]	Slovenia	Case series (4^b^)	TBI=3, brain tumor=1, and nonbrain injury=4	TBI or brain tumor: range 24-48 and nonbrain injury: range 27-40	Not reported	Not reported	Outpatient
Gamito et al [102]	Portugal	Case study	TBI=1 (severe)	20	Male	3 months	Inpatient rehabilitation ward
Ma et al [103]	United States	Case study^c^	TBI=1 (moderate-severe)	26	Male	9 months	Physical therapy clinic
Robitaille et al [112]	Canada	Proof of concept (4^b^)	TBI=6 (mild) and healthy controls=6	TBI: mean 30.3 (SD 8.6; range 18-61) and controls: mean 30.3 (SD 5.3)	Not reported	Median 0.46 years; range 2 weeks to 7 years	Not reported

^a^TBI: traumatic brain injury.

^b^Oxford levels of evidence.

^c^Conference proceeding.

#### Participant Characteristics

A total of 42 participants with TBI were included in this study (Table 2). The number of participants ranged from 1 to 31 (mean 8, SD 13). Time post injury ranged from 2 weeks to 7 years. Although not always reported, the majority of participants were males (25/42, 60%) aged between 18 and 61 years. Most of the included participants sustained a severe TBI (25/42, 60%) [102,111], followed by moderate TBI (7/42, 17%) [111], mild TBI (6/42, 14%) [112], and moderate-severe TBI (1/42, 2%) [103]. TBI severity was not reported for 3 participants [101]. Where reported, VR was used in inpatient [102] and outpatient settings [101,103,111].

#### Target of VR Assessment or Treatment

Impairments targeted in VR assessment included executive functions [112] and prospective memory [111]. VR treatments targeted attention and working memory [102], balance and functional mobility [103], and stress and anxiety [101].

#### VR Details

Keeping with the definition of immersive VR systems, all studies used HMDs to create immersive VEs [101-103,111,112], and one study included body motion tracking [112]. Virtual cities were used as the basis for memory and attention tasks [102,111]. Other VR tasks included a mindfulness-based stress reduction program [101], a military patrol task to assess executive functions [112], and standing balance practice in VR as an adjunct to traditional physical therapy [103].

Assessment studies did not report the time spent in VR. Where reported, therapy session duration ranged from 5 to 25 minutes; total dosage ranged from 50 minutes to 3 hours; and participants received 5, 8, or 10 therapy sessions. One study provided breaks during VR sessions [103]. Further information related to the VR details of the included studies is presented in Table 3.

**Table 3:** Virtual reality details of the studies included in part 2.

Study	Target	Dosage or time in VR^a^ and VR hardware	Task details	Outcome measures	Results	Adverse effects and potential issues	Eligibility criteria
Banville and Nolin [111]	Prospective memory and executive functions	Time in VR not reported; HMD^b^ (eMagin Z800) with head tracker	Non-VR task: prospective memory assessment based on Rivermead Behavioral Memory Test; VR task: virtual prospective memory tasks completed in a virtual city (included visiting apartments and selecting an apartment to live in)	Non-VR: correct actions, time to complete, and whether prompting was required; VR: prospective memory score, precision score, time to complete, success in task, and IPQ^c^	Participants could be classified as having a TBI^d^ by performance on each task. TBI participants were significantly less precise with prospective memory VR tasks (*P*=.02) and took more time to perform VR tasks (*P*=.008) than controls	SSQ^e^ completed; no reported cybersickness; SSQ scores did not differ between groups	Inclusion criteria: confirmed TBI
Cikajlo et al [101]	Stress and anxiety	8 sessions (25 minutes per session, once weekly); Samsung Galaxy X7 mobile phone mounted to HMD (Samsung Gear VR)	Mindfulness stress reduction program conducted by an instructor (eg, self-meditation and group discussions in various VEs^f^ such as a mountain view or a room with a fireplace)	MAAS,^g^ SWLS,^h^ MMSE^i^ (TBI only), session and task ratings, and head motion	Slight improvement in MAAS and SWLS scores (TBI group>non-TBI group); one participant increased MMSE (not reported for others); task ratings: simple to use and interesting; varying head motions	Potential for overheating of mobile phones (sessions were, therefore, limited to 30 minutes)	Inclusion criteria: mild or no cognitive impairment and able to understand instructions; exclusion criteria: high diopters, astigmatism, and wore glasses
Gamito et al [102]	Working memory and attention	10 sessions (5 minutes each session); HMD (eMagin Z800)	Activities included performing ADLs^j^ in the VE (eg, breakfast, navigating to and from a supermarket, and buying items)	PASAT^k^ and completion time of each task	Significant increase in correct responses between initial and final PASAT scores (*P*<.05)	Not reported	Inclusion criteria: diagnosed with a TBI 3-12 months prior, clinical deficit in memory and attention, and aged 18-60 years; exclusion criteria: a previous psychiatric disorder that may impact memory and attention and neurological diseases
Ma et al [103]	Balance and functional mobility	5 sessions (12 trials, with a 1- to 2-minute break in between); breaks were decided by the participant. Samsung Galaxy X7 mobile phone mounted to HMD (Samsung Gear VR)	Standing balance exercises in a VE with traffic lights, street crossing and traffic island, night and day versions, moving cars, and static buildings	DGI,^l^ mini-BEST,^m^ DHI,^n^ ABC,^o^ GROC,^p^ and patient-specific functional scale (self-scoring street crossing and multitasking abilities)	Improvements in DGI, mini-BEST, DHI, GROC, and patient-specific functional scale	Not reported	Not reported
Robitaille et al [112]	Executive functions	Time in VR not reported; HMD (piSight 166-43) with head tracking and body tracking (MoCap)	Exploration of a simulated military patrol scene in a village with different conditions and obstacles to navigate (eg, fences, wires, beams, and avatars)	PQ,^q^ SUS,^r^ errors, walking speed and fluidity, and obstacle clearance	TBI group walked faster and had slightly greater obstacle clearances. Significant difference in walking fluidity between groups for two hostile blocks (*P*=.046). Moderate to high presence scores on SUS and PQ	SSQ completed: 1 participant with TBI and 1 control participant reported slight headaches	Inclusion criteria (TBI): mild TBI; inclusion criteria (controls): no known TBI or other neurological or musculoskeletal issues

^a^VR: virtual reality.

^b^HMD: head-mounted display.

^c^IPQ: Igroup Presence Questionnaire.

^d^TBI: traumatic brain injury.

^e^SSQ: Simulator Sickness Questionnaire.

^f^VE: virtual environment.

^g^MAAS: Mindfulness Attention Awareness Scale.

^h^SWLS: Satisfaction With Life Scale.

^i^MMSE: Mini-Mental State Examination.

^j^ADL: activities of daily living.

^k^PASAT: Paced Auditory Serial Addition Task.

^l^DGI: Dynamic Gait Index.

^m^mini-BEST: Mini-Balance Evaluation System Test.

^n^DHI: Dizziness Handicap Index.

^o^ABC: Activities Balance Confidence Scale.

^p^GROC: Global Rating of Change.

^q^PQ: Presence Questionnaire.

^r^SUS: Slater-Usoh-Steed Questionnaire.

#### Comparisons

One assessment study investigated participant performance on a VR and non-VR assessment of prospective memory [111], with participants performing similarly on both tasks. Both assessment studies [111,112] included healthy control groups, with findings able to distinguish between participants with and without TBI. One intervention study [101] included unmatched participants without TBI and did not report major differences in outcomes between the groups.

#### Eligibility Criteria and Adverse Effects

One intervention study [101] excluded participants due to visual impairments that may have impacted their tolerance of the HMD. The Simulator Sickness Questionnaire [113] was used in 2 studies [111,112] to monitor potential adverse effects of VR, with only 1 participant with TBI reporting a slight headache [112]. A total of 2 studies did not consider or report adverse effects [102,103].

#### Outcome Measures and Results

Various outcome measures were used, and all studies included more than one measure. VR task-specific outcome measures were used in 4 studies [101,102,111,112]. A total of 4 studies used outcome measures traditionally used for non-VR tasks [101-103,111]. Depending on the outcome, intervention studies took measures pre-, mid-, and postintervention [101,102] or pre-post intervention with a 1-month follow-up [103].

The results from the assessment studies suggested that VR assessment tasks have the potential for use as novel diagnostic tools [111,112]. These studies included healthy controls and could classify participants as having a TBI or not by their performance on VR assessment tasks. The findings demonstrated a significant difference between groups for walking fluidity during a navigation task [112] and for time and precision to complete a procedural memory task in VR [111].

Statistically significant outcomes were reported in one case study [102], where VR intervention for attention and memory deficits led to a significant increase in pre-post assessment scores on the Paced Auditory Serial Addition Task. The remaining treatment studies presented descriptive data on the outcomes of participants with TBI [101,103]. Ma et al [103] demonstrated that VR treatment combined with standard physical therapy led to improvements in gait and balance. Cikajlo et al [101] reported slight improvements in pre-post psychometric outcomes for stress and anxiety following the implementation of a group-based VR mindfulness intervention that participants rated as interesting and simple to use.

#### Methodological Quality

The quality assessments of the included treatment studies are presented in Multimedia Appendix 3. We assessed 2 case studies using the JBI checklist for case reports [88] and these studies were found to have low [102] to moderate [103] methodological quality. The studies included adequate information about interventions but did not provide comprehensive participant or assessment details or refer to measuring the potential adverse effects of VR. One study [101] was appraised using the JBI checklist for case series [88] and had a low methodological quality. Information about condition measurement and treatment outcomes was provided, but important details about the participants and recruitment methods were absent.

#### Recommendations for VR: Evidence in TBI Studies

VR assessment and treatment studies for TBI rehabilitation were examined regarding the three suggested phases of VR development [66] and the nine categories of recommendations for VR design and implementation proposed in part 1 of this review (Table 4). With regard to phases of VR design, there was one feasibility study [101] and one proof-of-concept study [112] but no controlled trials or co-design studies with detailed descriptions of end user involvement in VR development. A total of 3 studies [101,111,112] have considered the potential adverse effects of VR. Rehabilitation principles were included in studies that provided varied or progressively challenging tasks [102,103]. All treatment studies collected at least one outcome measure pre- and postintervention and included clinically relevant outcome measures. At least one patient-reported outcome measure was included in 4 studies [101,103,111,112], and one study included a user survey for task feedback [101]. Details were generally provided about the *active ingredients* of the VR equipment and tasks (eg, dose, repetitions, and time in VR).

Recommendations that were not included in the assessment and treatment studies were as follows: involving researchers when developing VR tasks, considering barriers and facilitators to VR use, technological design and development, and supporting VR in practice. However, many of these recommendations are applicable to specific phases of VR development and implementation, so they may not have been relevant for all studies. Furthermore, recommendations may have been addressed but not specifically reported on.

**Table 4:** Inclusion of recommendations for virtual reality design and implementation in traumatic brain injury studies.

Recommendations for VR^a^ development			Banville and Nolin [111]		Cikajlo et al [101]		Gamito et al [102]		Ma et al [103]		Robitaille et al [112]
**Phase of VR development**											
	Phase 1: co-design										
	Phase 2: feasibility			✓^b^						✓	
	Phase 3: controlled trials										
**Recommendation**											
	End user involvement			✓							
	Participant factors										
	Adverse effects and safety	✓		✓						✓	
	Researcher involvement										
	Determining barriers and facilitators to VR										
	Rehabilitation principles					✓		✓			
	Technological design and development										
	Supporting implementation										
	Research study design, reporting, and analysis	✓		✓		✓		✓		✓	

^a^VR: virtual reality.

^b^Recommendation present.

## Discussion

### Principal Findings

#### Overview

The findings of this systematic review highlight that research in the field of VR and ABI rehabilitation, particularly for TBI, is still emerging. To our knowledge, this is the first study to synthesize existing recommendations for developing VR for ABI rehabilitation and to systematically review the current evidence base for using immersive VR for TBI rehabilitation. Recommendations for future research have been provided based on the results of this review.

#### Part 1

Part 1 of this review aimed to identify and synthesize the recommendations for designing and implementing therapeutic VR for ABI rehabilitation, with a focus on using existing frameworks to determine key technological and co-design factors. The findings appear to be consistent across VR technologies and health care settings and contain important considerations for using VR with people who have an ABI.

Three phases for VR development and implementation of therapeutic VR in health care were developed by Birckhead et al [66] and formed a framework against which this review was completed. A total of nine categories of recommendations were subsequently developed from all 14 studies included in part 1 of this review. Most recommendations that were addressed in the limited literature reported in this study were related to the design of VR tasks, including consideration of participant factors, involving key end users and researchers, determining barriers and facilitators of VR use, technological considerations, and including rehabilitation principles in VR tasks. The recommendations can be applied throughout VR development and implementation (Figure 2).

A phased approach to VR design should be considered [10,41,66], with early focus on engaging key end users [35,39,40,45,66,93,98,114] in co-design and feasibility studies before conducting larger controlled trials [10,41,66,115]. This approach is not widely used in research investigating VR for health care purposes [115-117] or for adults with TBI, yet user involvement in VR development is emerging in pediatric TBI [118] and ABI rehabilitation [98,99]. Involving end users in designing digital health interventions is recommended [66,114] and is essential for producing successful VR apps [40,66,67], particularly for people with TBI [119]. Additional recommendations for research design, reporting and analysis, and supporting implementation for VR were synthesized to further guide and strengthen research in this area. Such recommendations include the use of reporting guidelines such as the Template for Intervention Description and Replication [120] and the CONSORT (Consolidated Standards of Reporting Trials) [121] and supporting end users with VR adoption via education and training.

The included studies drew on research examining various VR systems and levels of immersion. This reflects the literature from the past decade and highlights the limited use of fully immersive VR for neurological rehabilitation. However, recommendations for VR research are similar across VR platforms, particularly for design and feasibility studies [66], and may be adapted for various clinical settings and disciplines [67]. No specific recommendations were made regarding preferred VR hardware or software. Future research and clinical VR apps should focus on more immersive systems [40] because of the rapid advancement and availability of VR technology as well as tasks that can be used across different devices to facilitate transition and use from rehabilitation facilities to home environments (eg, wireless systems and mobile phone compatibility) [39,122,123]. The tolerance and safety of new VR systems will need to be established for people with ABI [39-41,66,98].

#### Part 2

The second part of this review aimed to determine the current evidence base for using immersive VR for TBI rehabilitation and to review the extent to which these studies addressed the recommendations developed in part 1 of this review. A total of 5 studies that investigated the use of immersive VR for TBI assessment and treatment were identified and included.

The findings demonstrate a small body of evidence for using immersive VR in TBI rehabilitation. Studies have used immersive VR to assess cognitive impairment following mild, moderate, and severe TBI [111,112]. VR treatments targeted memory and attention [102] and balance [103] in single cases of participants with moderate-severe TBI and anxiety in a case series of 3 people with TBI (severity not disclosed) [101]. The range of time post injury, age of participants, clinical settings, dosage and frequency, and VR tasks suggest that immersive VR has potential for use with people with TBI across the continuum of care [68]; however, further studies are required to support this evidence because of the limited number of included studies and small sample sizes.

Three different HMDs were used in the 5 studies, including the smartphone-compatible Samsung Gear, which highlights the accessibility and affordability of immersive VR technology [80,115,123]. There were no commonalities in terms of VR software and tasks, with each study implementing VR tasks specific to the targeted impairments and outcomes. There would be potential challenges in developing a VR platform to suit the various impairments and severities of brain injury [95], so the proposed recommendations developed in part 1 of this review may improve the consistency of VR development for TBI rehabilitation where heterogeneity may not be accounted for.

There were limited adverse effects of VR use reported in 3 of the 5 included studies [101,111,112]. The potential adverse effects of VR use must be monitored and reported due to the limited research in this area and to determine the safety and feasibility of immersive VR for people with TBI [39-41,66]. Involving VR experts and interdisciplinary teams should be considered when designing new VR tasks to mitigate potential safety issues [32].

The included studies reported positive findings, but few specific conclusions can be drawn regarding assessment and treatment effectiveness due to a limited number of studies with small sample sizes, a lack of control conditions, assessment reference standards, face-to-face comparisons, and heterogeneity of data that prevented pooling of data and meta-analysis. Some studies had relatively low methodological quality and provided minimal details about participants and recruitment methods, making it challenging to generalize findings and determine the suitability of VR platforms and tasks for people with TBI.

The current evidence base for using immersive VR for TBI rehabilitation incorporates some of the recommendations proposed in part 1 of this review (Table 4), yet there is a need for future studies to increase end user engagement in co-design and feasibility testing before conducting controlled trials [10,41,66]. Specifically, future research should engage end users [39,40,66,67,95] and clinical experts alongside the design and technology industries to inform VR development [35,39,95], identify potential barriers and facilitators to using VR [35,39,66,67], and focus on stepwise progression of VR research [10,41,66,114]. By doing so, VR tasks for TBI, and ABI rehabilitation more broadly, can better meet patients’ and therapists’ needs [35,39,40,66,67].

### Study Limitations

Although a systematic literature search was undertaken, some existing studies may have been excluded, as inclusion criteria limited papers to English only, and gray literature did not include conference abstracts or theses. In addition, inconsistencies with VR definitions and classifications [124-127] may have led to exclusion based on the definitions of VR immersion levels. Caution should be taken when interpreting results, as the overall levels of evidence presented were relatively low. Furthermore, the inclusion of various study designs led to the inability to use a single critical appraisal tool, and some of the included studies presented poor methodological quality.

There were limited high-level evidence studies that provided recommendations for developing and implementing VR in ABI rehabilitation (ie, part 1). Although this may decrease the perceived value of findings, it likely reflects the fact that VR technology and practice in this field are still emerging [35] and may be moving faster than the evidence base [66,93]. Despite this, most recommendations were synthesized from a VR expert consensus paper [66], which provided a framework for therapeutic VR methodology. Furthermore, findings across the included studies were similar and provide a basis for ongoing research for developing and implementing VR for ABI rehabilitation, including for people with TBI.

The current evidence base for using immersive VR for TBI assessment and treatment (ie, part 2) consisted mainly of lower-quality methods of case studies and case series. These study designs may be suitable for early co-design and feasibility studies for VR development, yet this was not always reflected in the included studies. On the basis of the methodological quality and levels of evidence, future studies should provide important details about participants, recruitment methods, and interventions; consider and report on adverse effects; and include reference standards and control conditions. These findings reflect the general lack of high-quality evidence, as highlighted in previous reviews of nonimmersive, semi-immersive, and immersive VR for TBI rehabilitation [34,56,68,70,71], and the findings may be difficult to generalize due to heterogeneity. The use of the proposed recommendations may improve the consistency of design and implementation of VR in TBI rehabilitation and provide a model to advance clinical research in this area.

### Recommendations for Future Research

Future research should consider the proposed recommendations when designing and implementing VR tasks for ABI rehabilitation, especially for people with TBIs. As identified in this review, stepwise VR development (Figure 2) [10,41,66] is lacking in current TBI literature. There needs to be an increased focus on co-design processes to investigate the opinions and needs of key end users, including people with ABI and their therapists [10,35,39,41,66,67,114]. Iterative testing and feasibility studies will also be necessary to establish the safety and viability of new immersive VR tasks before implementing larger-scale studies and RCTs [10,41,66], particularly given that people with ABIs may face challenges when using and interacting with VR systems [39,40] and there are limited studies that use immersive VR for TBI rehabilitation [101-103,111,112].

Although this review offers a starting point for guiding future research in VR for TBI rehabilitation, the recommendations provided were formed from papers that included a wider range of ABIs. Work should be undertaken to develop guidelines specific to TBI to ensure more rigorous development and evaluation of therapeutic VR for this population. Expanding the evidence base for using VR with people with TBI has been encouraged and highlighted as a priority area in published guidelines for TBI management [53,104,105]. Furthermore, immersive VR to date has been used to treat people with TBI who have cognitive disorders, balance issues, or anxiety. Future research should develop and investigate the use of VR for other significant impairments that people with TBI may experience, such as cognitive-communication disorders.

### Conclusions

This systematic review highlights that the use of immersive VR in ABI rehabilitation, especially TBI, is still in its infancy. There are no existing guidelines for designing and implementing VR tasks specific to TBI, reflecting the need for more rigorous research in this area. Existing evidence demonstrates the potential to use immersive VR for TBI assessment and treatment. However, this comprises a small number of lower-quality studies with a large degree of heterogeneity, small sample sizes, and limited generalizability of the findings.

This review produced recommendations for developing and implementing VR for ABI rehabilitation (Textbox 2 and Multimedia Appendix 2): engaging end users; considering participant, researcher, and technological factors; addressing facilitators and barriers; incorporating rehabilitation principles; and supporting implementation in clinical practice. These recommendations can be incorporated into the three phases of therapeutic VR development (Figure 2). Researchers in ABI rehabilitation are presented with an opportunity to capitalize on the current digital health movement, particularly when the required technology and resources for immersive VR are becoming increasingly available and affordable. VR has the potential to provide innovative assessment and treatment methods, and future work in this field should use these recommendations to improve consistency, quality, and outcomes for the effective design of therapeutic VR.

## Appendix

Multimedia Appendix 1 Example database searches.

Multimedia Appendix 2 List of recommendations for the design and implementation of virtual reality for acquired brain injury rehabilitation.

Multimedia Appendix 3 Quality appraisal of the included studies.

## Abbreviations

ABI: acquired brain injury
CONSORT: Consolidated Standards of Reporting Trials
HMD: head-mounted display
JBI: Joanna Briggs Institute
PRISMA: Preferred Reporting Items for Systematic Reviews and Meta-Analyses
RCT: randomized controlled trial
TBI: traumatic brain injury
VE: virtual environment
VR: virtual reality
