# Supplementary material for: Recommendations for the Design and Implementation of Virtual Reality for Acquired Brain Injury Rehabilitation: Systematic Review
Source: J Med Internet Res. 2021 Jul 30;23(7):e26344. doi: 10.2196/26344 (PMC8367177; doi:10.2196/26344)
Supplement: Multimedia Appendix 1 [file jmir_v23i7e26344_app1.pdf]

Example database searches

Medline search for Part 1

Step	*Key word*
1	exp Virtual Reality/
2	virtual reality.tw.
3	1 or 2
4	exp Guideline/
5	(guideline* or practice guideline* or framework).tw.
6	4 or 5
7	exp Clinical Trial/
8	(assess* or evaluati* or diagnos* or intervention* or therapy or treat* or trial* or clinical practice).tw.
9	7 or 8
10	exp Rehabilitation/ or exp brain injuries/ or exp Head Injuries, Closed/
11	(rehabilitation or neurorehabilitation or neurolog* or traumatic brain injur* or brain injur* or TBI or ABI or closed-head injur*).tw.
12	10 or 11
13	3 and 6 and 9 and 12
14	limit 13 to english language
15	limit 14 to yr="2009 -Current"

Medline search for Part 2

Step	*Key word*
1	exp virtual reality/
2	virtual reality.tw.
3	1 or 2
4	exp brain injuries/
5	exp Head Injuries, Closed/
6	(traumatic brain injur* or TBI or brain injur* or closed head injur*).tw.
7	4 or 5 or 6
8	(assess* or evaluat* or diagnos* or intervention* or rehabilitation or therapy or treat* or trial*).tw.
9	3 and 7 and 8
10	limit 9 to english language
11	limit 10 to yr="2009 -Current"

This is a Multimedia Appendix to a full manuscript. For full copyright and citation information see http://dx.doi.org/10.2196/jmir.26344
