# Supplementary material for: Recommendations for the Design and Implementation of Virtual Reality for Acquired Brain Injury Rehabilitation: Systematic Review
Source: J Med Internet Res. 2021 Jul 30;23(7):e26344. doi: 10.2196/26344 (PMC8367177; doi:10.2196/26344)
Supplement: Multimedia Appendix 2 [file jmir_v23i7e26344_app2.pdf]

## Recommendations for the Design and Implementation of Virtual Reality for Acquired Brain Injury Rehabilitation

### 1. End user involvement

1.1 Involve end users during VR development and implementation [39,40,66,67,95,98]:

- 1.1.1 Conduct iterative testing with VR prototypes and gather positive/negative feedback from intended end users.
- 1.1.2 Conduct observations, questionnaires, interviews, or focus groups to establish end user opinions, needs, challenges, and expectations (eg, technology experience, co-morbidities) [66].
- 1.1.3 Document all feedback from end users [66].

### 2. Participant factors

2.1 Define end users and how they will experience VR in the context of their condition(s) [66].

2.2 Consider a range of participant factors when designing VR tasks (eg, gender, age, health conditions, cognitive/physical/communication impairments) [40,66,95-98].

2.3 Observe intended users in a clinically relevant or specific context to learn about their behavior(s) [66].

2.4 Examine the impact of VR on patient motivation and engagement in rehabilitation [10,66,98].

### 3. Adverse effects and safety

3.1 Measure and describe patient-reported adverse effects associated with VR tasks and equipment. This should be documented for healthy users and the intended patient population(s) [40,66].

3.2 Examine the safety of VR devices and tasks to determine suitability/contraindications for a given population [39,41,98].

### 4. Researcher involvement

4.1 Develop ideas for testing as a team [66].

4.2 Evaluate and develop prototypes for end user testing based on ideas that are feasible (eg, within financial limitations) [66].

### 5. Determining barriers and facilitators to VR

5.1 Identify potential barriers and facilitators to designing and implementing VR with key stakeholders [35,39,66,67]:

- 5.1.1 Include patient, therapist, administrator, technical, site-specific, and operational facilitators and barriers to use.
- 5.1.2 Consider facilitators and barriers to location, personnel for support, costs, client motivation, and therapist experience.

5.2 Identify causes of barriers and offer solutions or implementation strategies [35,66].

## 6. Rehabilitation principles

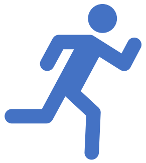

6.1 Maintain therapeutic principles in VR task designs (eg, principles of motor learning) [94,95].

6.2 Provide tasks that can be gradually modified to progressively challenge physical and/or cognitive ability in line with abilities of end users (consider condition severity and stage of recovery) [94-98].

6.3 Feedback:

- 6.3.1 Provide feedback of performance in real-time to sustain engagement and motivation [94-96].
- 6.3.2 Provision of knowledge of performance/results should not interfere with task performance [94-96].
- 6.3.3 Consider providing multimodal feedback (eg, visual, auditory, haptic) [94,96,97].
- 6.3.4 Feedback modality should consider cognitive level/performance [94-97].

## 7. Technological design and development

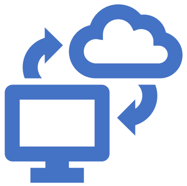

7.1 Use ‘technically sound’ and ‘flexible’ hardware and software [95]:

- 7.1.1 Avoid complex or restrictive interfaces.
- 7.1.2 Ensure that hardware allows for adequate movement and considers postural constraints.

7.2 Work in collaboration with VR experts, game developers and/or engineers [35,39,40].

## 8. Supporting implementation

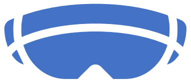

8.1 Support therapists with VR adoption:

- 8.1.1 Facilitate time for learning (consider train-the-trainer or mentoring models) [35,39,67].
- 8.1.2 Identify ways to support engagement, training, and troubleshooting [67,93,99].
- 8.1.3 Provide clear information about ways to use VR for rehabilitation and how to maximize user engagement [35,39,67,98,99].
- 8.1.4 Ensure access to technical supports and systems (eg, adequate internet access, troubleshooting) [39,99].

8.2 Provide education and continued training for therapists and students [35,39,93]:

- 8.2.1 Include tailored clinical training packages and tools based on best practices and therapist needs.

8.3 Provide information, training, and support for patients using VR in terms of familiarization, purposes of using VR, adequate instructions, and monitoring of performance (either in the clinic or remotely) [95,96,98,99].

## 9. Research study design, reporting, and analysis

9.1 RCTs:

- 9.1.1 Conduct large, adequately powered trials [10].
- 9.1.2 Use appropriate methods for randomization (eg, computer generation) [66].
- 9.1.3 Ensure that allocation is concealed [66].
- 9.1.4 Select and justify control condition(s) [10,66].
- 9.1.5 Control interventions should be described and standardized [93].

9.2 Reporting:

- 9.2.1 Describe intervention details (eg, dose, frequency, repetitions, intensity, equipment) [41,66] and consider using gudelines for reporting of interventions (eg, TIDIER, CONSORT) [66].
- 9.2.2 Report on methods of concealment and blinding [66].
- 9.2.3 Provide information about the number of participants screened against eligibility criteria to provide information about who may be suitable to use VR [10, 66].
- 9.2.4 Register trials and publish all research regardless of the outcome(s) [66].

9.3 Analysis [66]:

- 9.3.1 Employ intention-to-treat analysis for primary outcome measures.
- 9.3.2 If relevant, report on per-protocol analysis.
- 9.3.3 Aim for a minimum of 10 observations for each independent variable in multivariable analyses.

9.4 Outcome Measures:

- 9.4.1 Outcomes should be clinically relevant [41,66], validated [66], and common to increase usefulness of meta-analysis [10, 93].
- 9.4.2 Consider using patient-reported outcome measures [41,66].
- 9.4.3 Outcome measures should be taken pre- and post-VR intervention [10,66].
- 9.4.4 Modify or revalidate psychometric properties if needed (in the context of immersive VR) [41,66].
- 9.4.5 Measure the long-term effects of VR interventions (ie, at least three months postintervention) [10,66].
- 9.4.6 If possible, use control groups for comparison of outcomes [66].
- 9.4.7 Future research on participation outcomes should evaluate VR practice within natural environments [93].

This is a Multimedia Appendix to a full manuscript. For full copyright and citation information see http://dx.doi.org/10.2196/jmir.26344
